# From self-regulated learning to computer-delivered integrated speaking testing: Does monitoring always monitor?

**DOI:** 10.3389/fpsyg.2023.1028754

**Published:** 2023-02-01

**Authors:** Weiwei Zhang, Aaron Wilson

**Affiliations:** ^1^School of Foreign Languages and International Education, Quzhou University, Quzhou, China; ^2^School of Curriculum and Pedagogy, Faculty of Education and Social Work, University of Auckland, Auckland, New Zealand

**Keywords:** self-monitoring, self-regulated learning, speech production, computer-delivered integrated speaking testing, L2 learners

## Abstract

Despite the salience of monitoring in self-regulated learning (SRL) and foreign and/or second language (L2) speech production in non-testing conditions, little is known about the metacognitive construct in testing contexts and its effects on learner performance. Given the reciprocal effects between L2 testing and L2 learning, a research effort in monitoring working in speaking tests, in particular computer-delivered integrated speaking tests, a testing format that has been advocated as an internal part of L2 classroom instruction and represents the future direction of L2 testing, is warranted. This study, therefore, serves as such an effort through investigating the use of monitoring by 95 Chinese English as foreign language (EFL) learners on a self-reported questionnaire after they performed three computer-delivered integrated speaking test tasks. Descriptive analysis followed by Hierarchical Linear Modelling (HLM) testing reveals that monitoring was used in a high-frequency manner, but it exerted no substantial effects on learner performance. Primarily, the results are expected to provide pedagogical implications for SRL: while fostering self-regulating learners, especially self-monitoring L2 speakers, it is necessary for L2 teachers to purposefully reduplicate testing conditions in their classroom instructions for helping the self-regulating learners be equally self-regulating test-takers. Moreover, the results are hoped to offer some insights into L2 testing through the perspective of self-monitoring, one proposed component of strategic competence, a construct that has been extensively acknowledged to reflect the essence of L2 testing.

## Introduction

“Both language tests and the English language play powerful roles in today’s world and that the combination of these two powerful entities has far reaching implications for policy and practice in English language teaching” (Shohamy, 2007, p. 1). As long as 15 years ago, Elana Shohamy, a well-known scholar of language testing use, identified the powerful influence of L2 testing on L2 teaching. Shohamy’s finding still holds true today, and many empirical studies have evidenced the power of L2 testing as a gate-keeper in judging the quality of a certain pedagogical practice in L2 teaching (e.g., Huang et al., 2016). Built upon the evidence, this present article put self-monitoring, the core skill of SRL, under the microscope of L2 testing, specifically, the computer-delivered L2 integrated speaking testing as rationalized subsequently, to investigate if the self-regulatory skill works in testing conditions. The investigation is expected to provide pedagogical implications for SRL: while fostering self-regulating learners, it is necessary for L2 teachers to purposefully reduplicate testing conditions in their classroom instructions for helping the self-regulating learners be equally self-regulating test-takers assisted by self-monitoring to pass certain high-stakes tests for either academic purposes or vocational objectives, the fundamental goal of L2 learning for a large proportion of L2 learners (Shohamy, 2007; Bachman and Palmer, 2010). Moreover, the investigation is hoped to offer some insights into L2 testing through the perspective of self-monitoring, one proposed key component of strategic competence, a construct that has been extensively acknowledged to reflect the essence of L2 testing (Bachman and Palmer, 2010; Zhang et al., 2021a, 2022a,b).

In the research domain of L2 teaching, SRL is seen as one of the most effective tools to cultivate self-regulating learners who are competent enough to set their own learning goals independently and metacognitively monitor their learning progress toward achieving these goals (Zimmerman, 1986; Schunk and Greene, 2018; Zhang and Zhang, 2019; Teng, 2022). Although it is unclear when systematic explorations of SRL began (Schunk and Greene, 2018), it is widely believed that the first papers (e.g., Zimmerman, 1989; Pintrich et al., 1993) distinguishing SRL from metacognition published in 1980s elicited waves of studies that have explored the concept from diverse theoretical perspectives: social-cognitive, cognitive/metacognitive which is also termed information-processing, developmental, motivation and emotion, and co-regulation and socially shared regulation. These diverse perspectives share one evident commonality: self-monitoring plays a paramount and indispensable role in SRL, and self-regulating learners are most commonly characterized by their active and efficient management of learning through self-monitoring (e.g., Greene and Azevedo, 2007; Griffin et al., 2013; DiBenedetto, 2018; Schunk and Greene, 2018).

Similarly, in the research field of L2 speaking, self-monitoring is acknowledged to work both covertly and overtly, considerably affecting L2 speech production (Kormos, 2006, 2011; Bygate, 2011). Some scholars (e.g., Uztosun, 2021; Gan et al., 2022) have proposed that SRL effectively assists L2 learners to surmount the difficulty of acquiring L2 speaking, a daunting task assumed by a vast majority of L2 learners (Uztosun, 2021; Gan et al., 2022). Despite the intimate relationship between SRL and L2 speaking, scant literature is available that contextualizes SRL in L2 speaking or vice versa (Uztosun, 2021; Gan et al., 2022), in particular from the angle of self-monitoring, although a large body of research has been done to investigate SRL in the contexts of writing (e.g., Teng and Zhang, 2020), listening (e.g., Vandergrift and Goh, 2012), and reading (e.g., Cirino et al., 2017). In addition to lack of relevant literature, the salience of speaking also served as our motivation to conduct the present study: speaking is the direct means through which communications in real-world settings take place (Luoma, 2004).

Further, our focus on self-monitoring in the context of L2 speaking testing, specifically in computer-delivered L2 testing is due to the ever-increasing prevalence of the testing format as a result of technological evolution, in tandem with COVID-19 which has imposed a great challenge to traditional off-line teaching activities and called for virtual learning with assistance of computers (Zhang et al., 2021a). In accordance with our research motivations and focus, we adopted a multi-disciplinary perspective involving SRL, L2 speaking, computer-delivered testing, and integrated language skills in studying into if and how self-monitoring, the core SRL skill (Winne and Hadwin, 1998; Winne, 2011, 2018), works in the context of computer-delivered integrated L2 speaking tests.

For a clear and general understanding of the roles that self-monitoring plays in the above-mentioned disciplines theoretically and empirically so as to evidence the importance of the construct and accordingly the necessity of additional research efforts, such as our study, we first conducted a comprehensive review of literature concerning the roles of self-monitoring across these disciplines. During the review, we set a special focus on prior empirical studies into how monitoring works in L2 testing conditions, in particular computer-delivered integrated L2 testing, which helped to formulate the research questions of this study. Following the review, we reported our empirical study and discussed its contributions to SRL in relation to L2 speaking and L2 testing before providing some suggestions for future studies of relevance. It has to be pointed out that self-monitoring is the operational form of metacognitive monitoring, or in short terms, monitoring, in SRL and speaking (e.g., Levelt, 1983, 1989; Zimmerman, 2000; Kormos, 2006, 2011; Bygate, 2011; Schmitz and Perels, 2011; Nozari, 2020; Teng, 2022), and thus, we used “self-monitoring,” “metacognitive monitoring” and “monitoring” interchangeably in this article.

## Monitoring in SRL

Since its inception, the concept of SRL has been researched from multiple perspectives (see Panadero, 2017, for a review), and hence the definitions of the concept differ, which is illustrated by various SRL models, such as Zimmerman’s (1989) social-cognitive model of SRL that has been widely used in the prior studies (Panadero, 2017; DiBenedetto, 2018; Schunk and Greene, 2018). Regardless, we explored the role of self-monitoring in SRL from the perspective of information-processing incarnated by Winne and Hadwin’s (1998) SRL model. We did this for the consistency across the disciplines involved in this study because both L2 speaking and L2 speaking testing are recognized as an information-processing procedure (Hughes and Reed, 2017; Yahya, 2019). In addition, Winne and Hadwin’s (1998) model has been acknowledged as one of the most applied and cited SRL models in research where SRL is implemented for computer supported learning (Panadero, 2017; DiBenedetto, 2018; Schunk and Greene, 2018).

**Figure 1:**
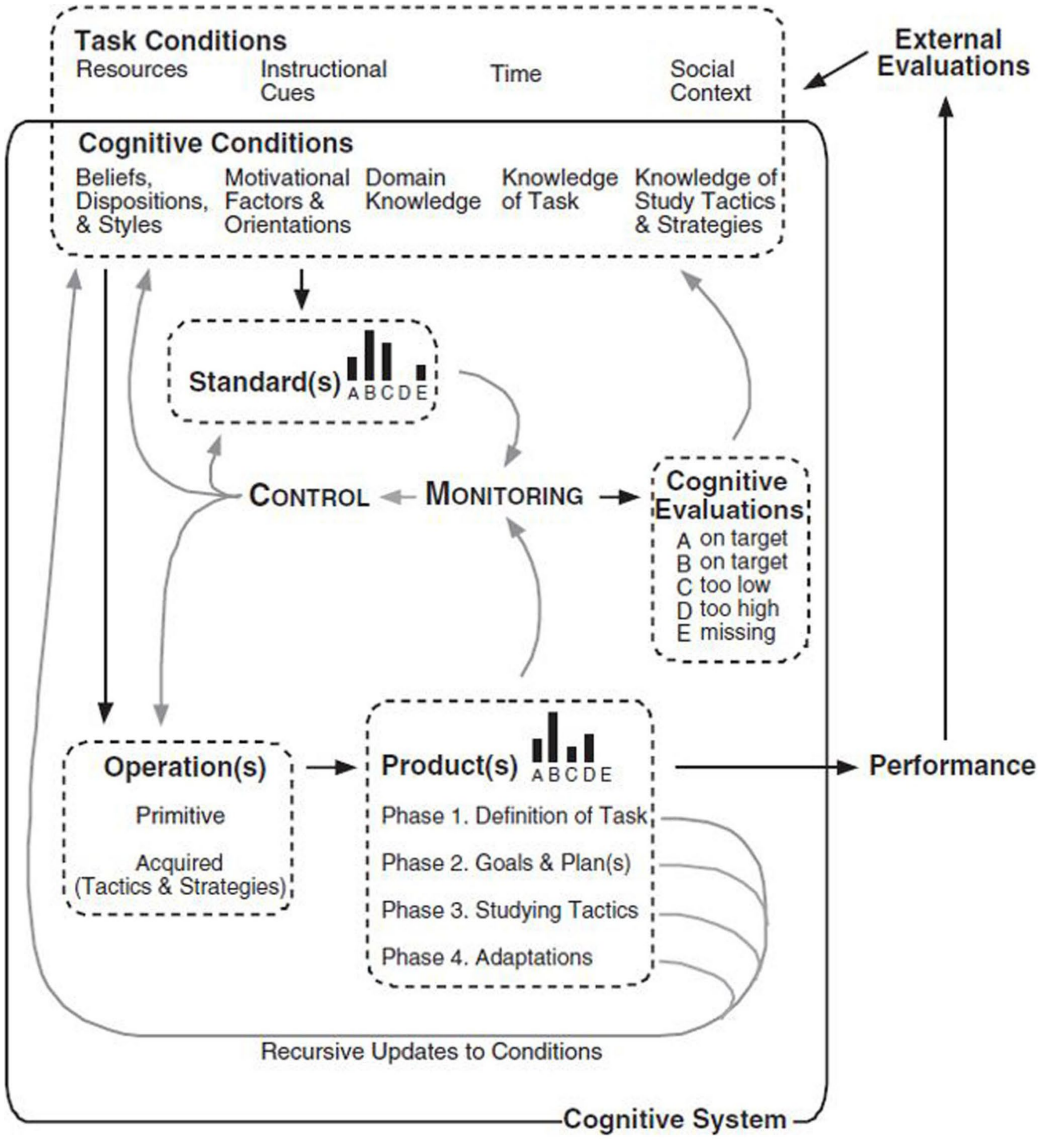
Winne and Hadwin’s (1998) model of self-regulated learning from Perry and Winne (2006) learning from Learning Kits: gStudy Traces of Students Self-Regulated Engagement with Computerized Content (p. 213). *Educational Psychology Review*, 18, 211–228.

In Winne and Hadwin’s (1998) model, monitoring is of central importance because the construct and metacognitive control enacted in line with monitoring serve as the two events that form the hub of SRL which is divided into four phases: task definition, goal setting and planning, tactics enactment, and metacognition adaption. In the actual process of SRL, each of these four phases is proposed to be carried out through the interactions of the five elements or task facets covering conditions, operations, products, evaluations and standards acronymized by Winne and Hadwin (1998) as COPES (Panadero, 2017), as illustrated in Figure 1.

Figure 1 shows that the five task facets, except for operations, are all treated as information in one form or another. Conditions refer to resources or constraints that L2 learners perceive as related to their learning, which accommodate task conditions and cognitive conditions. Task conditions are L2 learners’ perceptions of tasks in accordance with outside environment such as instruction cues from their teachers in giving assignment and time that can be allocated for learning, while cognitive conditions relate to the information that L2 learners retrieve from their long-term memory, including their prior knowledge on the tasks they are going to perform and on study tactics and strategies that they believe they can use for performing the tasks. Motivational factors also influence the cognitive conditions. On the other hand, operations indicate the actual information processing that occurs in every phase, which, according to Winne and Hadwin (1998), and Winne (2001, 2011, 2018), comprise five cognitive operations, including searching, monitoring, assembling, rehearsing and translating (SMART), and they work at an object level and a meta-level. A product is the new information generated when SMART manipulates the available information, and the outcome of each phase can be treated as a product, which is built toward the goal of task completion in the form of performance. Standards denote the assumed qualities of products, or “the optimal end of whatever phase is currently running” (Greene and Azevedo, 2007, p. 336). Generally, standards originate from learning objectives given by teachers or by authors of textbooks, and from L2 learner’s own knowledge based on their memories of prior performance in similar tasks and expectations about their future performance regarding level and quality (Winne, 2018). The comparison by L2 learners between products and standards through monitoring creates cognitive evaluations, the fifth element in the SRL model. When a mismatch between products and standards occurs, L2 learners’ metacognitive control will be enacted. Learners will control their learning operations to refine the products and revise the conditions and standards, or revise the two facets simultaneously. Through the control over products and standards, a match between the two facets will be achieved, which guarantees the achievement of the learners’ learning goals (Greene and Azevedo, 2007; Schunk and Greene, 2018; Winne, 2018).

As delineated earlier, the five aspects interact with one another, executing the work of every phase of SRL. Specifically, in the first phase of task definition, L2 learners examine the conditions represented by resources available and constraints that may impede their L2 learning, such as time and information accessible, their personal interest and relevant knowledge in order to define a task. In this phase, learners will develop their perceptions of tasks and may use the standards or their prior knowledge and instructions from their teachers to monitor if their perceptions are appropriate. The product of task definition based on learners’ task perceptions will lay a foundation for the second phase of goal setting and planning where L2 learners set their specific goals for learning in light of their definitions of tasks. In essence, these goals serve as a set of standards that self-regulating learners will use to metacognitively monitor their progress during the whole procedure of learning and the final products of learning. By referring to these goals, L2 learners forge their plans, which can be revised as they proceed through their learning to approach these goals. In the third phase of tactics enactment, L2 learners begin their learning by enacting study tactics and strategies during which learners will compare the product and the process of learning respectively against the goals and plans set in the second phase. If mismatch is identified, metacognitive adaptions through learners’ control over conditions operations and plans for future learning will occur in the final optional phase of adaptions (Winne and Hadwin, 1998; Greene and Azevedo, 2007; Schunk and Greene, 2018; Winne, 2018).

The four phases of SRL are posited to be loosely and recursively sequenced, which permits L2 learners to move flexibly across phases rather than in a linear sequence in which products are sequentially updated from previous phases (Winne and Hadwin, 1998; Greene and Azevedo, 2007; Schunk and Greene, 2018; Winne, 2018). To exemplify the loose and recursive process of SRL, imagine a learner who is searching for tactics or strategies to tackle the task in the phase of enactment: if the learner is a self-regulating learner, she may move back to task definition in the first phase, inspecting and monitoring task conditions again for more useful resources. Likewise, when a self-regulating learner finds, through monitoring, that the tasks she is expected to perform have a lot of in common with those she has practiced multiple times before, the self-regulating learner may skip the first phase of task definition and go directly to the second phase of goal setting and planning. Similarly, a self-regulating learner may oscillate frequently between the phase of goal setting and planning and the phase of enactment after monitoring for a quick product. Also, metacognitive adaption through metacognitive control can take place at any point of SRL as a result of monitoring (Winne and Hadwin, 1998; Greene and Azevedo, 2007; Winne, 2018).

From the working schema of Winne and Hadwin’s (1998) model of SRL, it can be seen that the constant comparison between products and standards through monitoring makes SRL possible; and the continuous monitoring allows a learner to migrate across the phases of SRL in a recursive manner for learning efficiency and effectiveness. In short, it is metacognitive monitoring that plays a critical part in empowering a learner to be a self-regulating learner.

## Monitoring in l2 speech production

It is known that speaking, in particular, L2 speaking is a very complicated productive skill (Levelt, 1989; Yahya, 2019; Newton and Nation, 2020). Speaking is “a process of oral language production” (Tarone, 2005, p. 485) in which information is received and processed before systematic utterances are produced to express meaning that occurs in the real time situations (Levelt, 1989; Levelt et al., 1999; Yahya, 2019). Therefore, in the research discipline of speaking, whether it be L1 speaking or L2 speaking, speech production is typically treated as an information-processing procedure (Luoma, 2004; Bygate, 2011; Kormos, 2011; Zhang et al., 2022a,b) where monitoring plays a necessary role (e.g., Levelt, 1989; Levelt et al., 1999; Nozari and Novick, 2017; Broos et al., 2019), as it does in SRL.

**Figure 2:**
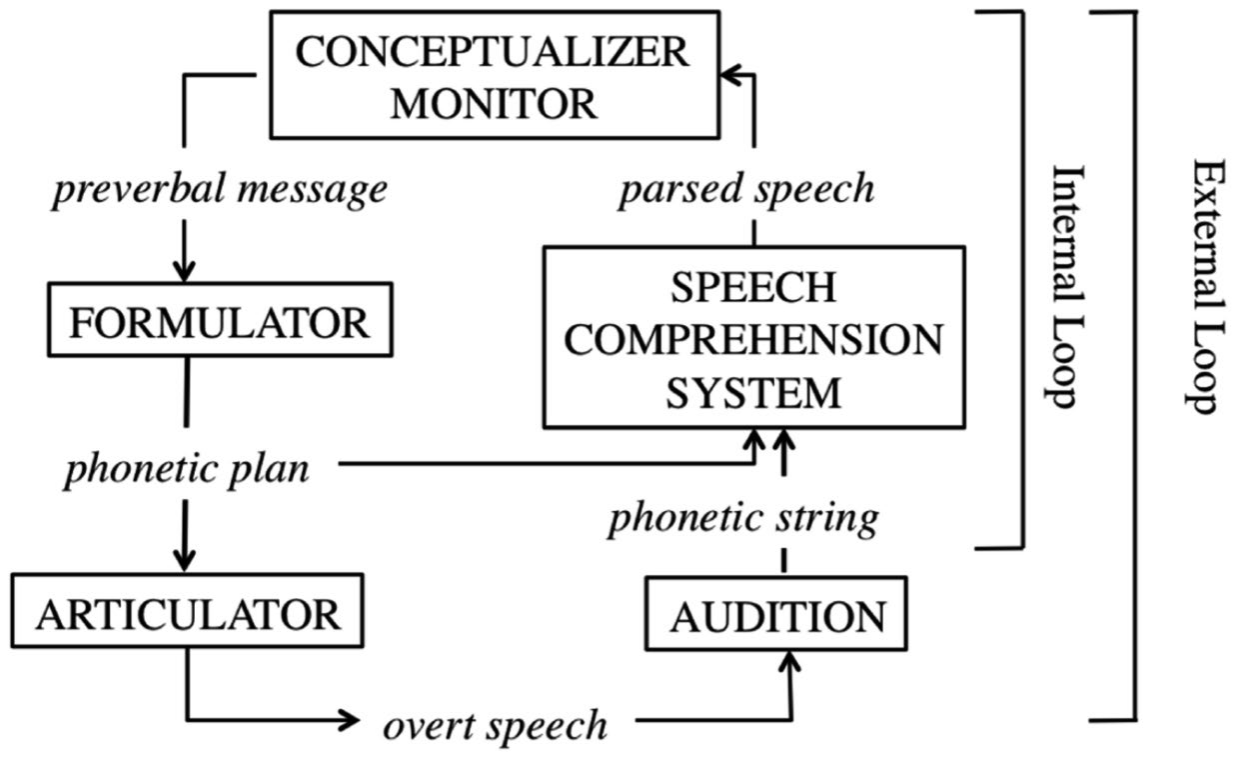
Levelt’s (1989) perceptual loop model of self-monitoring from Gauvin and Hartsuiker (2020). Towards a new model of verbal monitoring (p. 4). *Journal of Cognition*, 3(1).

In speaking, speakers are information processors, and “monitor what they are saying and how they are saying it” (Levelt, 1989, p. 458). Through such monitoring, errors, inappropriateness and other problems can be identified and accordingly corrected and adjusted (Levelt, 1989; Levelt et al., 1999). Alternatively stated, self-monitoring ensures that speakers’ utterances do reflect their communicative intentions, and in the meanwhile conform to linguistic standards through error detection and self-repair (Levelt, 1989; Levelt et al., 1999; Nozari and Novick, 2017; Broos et al., 2019). To duplicate the mechanism of monitoring in speech production, researchers have proposed four main approaches: comprehension-based monitoring, comprehension-perception based monitoring, production-based monitoring and forward models of monitoring (see Nozari and Novick, 2017; Nozari, 2020, for a review). Under the umbrella of these approaches are models of self-monitoring in speech production (see Nozari and Novick, 2017; Nozari, 2020, for a review) among which Levelt’s (1983, 1989) perceptual loop model of self-monitoring shown by Figure 2 is regarded as the most prominent and highly long-standing model of self-monitoring in speech production (Nooteboom and Quené, 2017; Gauvin and Hartsuiker, 2020; Nozari, 2020).

As seen in Figure 2, during speaking, a speaker’s utterances are generated through conceptualizer which constructs the preverbal message that needs to be expressed in language production in accordance with the speaker’s communicative intentions, and formulator where the preverbal message is linguistically encoded, including lexical retrieval, and grammatical and phonological encoding for a phonetic plan or internal/covert speech that will enter articulator where the phonetic plan is executed and transformed into overt speech. The audition in charge of auditory processing of speech sounds will map the overt speech to a phonetic string from which the speech comprehension system generates parsed speech. According to Levelt (1983, 1989), speech production, as a whole, is essentially a feedback system, and so the final output of the speech production, the parsed speech, will go back to the conceptualizer where it will be compared with the speaker’s communicative intentions from the aspect of phonological, morphological, syntactic and semantic composition through self-monitoring *via* a monitor located in the conceptualizer.

During the whole speech production, two loops of self-monitoring operate in the process, detecting errors and making relevant self-repair to ensure the correctness and appropriateness of the speech that is intended to be produced. The first loop is the internal loop that relates to the speaker’s inner or covert speech, monitoring the phonetic plan composed of grammatical and phonological codes of an utterance before it is actually pronounced or articulated. The second loop is labelled as the external loop, or the auditory loop (Oomen and Postma, 2001; Ganushchak and Schiller, 2006) which assumes the responsibility of monitoring speakers overt speech based on their auditory perception of the external speech. In fact, some scholars (e.g., Hartsuiker and Kolk, 2001; Oomen and Postma, 2001; Kormos, 2006, 2011) postulated the third self-monitoring loop in addition to the above-mentioned two loops: the conceptual loop comparing the preverbal messages and speaker’s initial communicative intentions and determining if specific vocabularies and expressions are appropriate for a particular context. Our understanding is that although the function of the conceptual loop has been included in the two loops, the extraction of the conceptual loop as an independent third loop is necessary as it indicates the equal salience of appropriateness and error detection as a result of self-monitoring. A concrete example of appropriateness monitoring comes from Hartsuiker and Kolk (2001) where “a glass” is not seen as an error, but is not as appropriate as “a tall glass” in a certain context. In this sense, the three-loop proposal is a more complete depiction of self-monitoring in speech production.

Regardless, the two or the three loops of self-monitoring are both proposed to be built upon the single speech comprehension system: speakers monitor their covert and overt speech based on their comprehension of the speech, of the contexts where the speech is going to be delivered, and of their intentions of delivering the speech in line with task demands. It is due to the single comprehension system that speakers can utilize “the same cognitive machinery for monitoring their own speech and for the speech of other people” (Hartsuiker, 2007, p. 95) in actual communications, such as monitoring their own speech in response to the interlocutors’ background knowledge, feedback, and the common ground between the speakers and the interlocutors (Nozari, 2020).

The self-monitoring loops are closely related to the comprehension system, which explains why self-monitoring in Levelt’s (1983, 1989) perceptual loop model is conceptualized under the category of the comprehension-based monitoring. New models have been proposed as understanding of self-monitoring in speech production has deepened. For instance, based on Levelt’s (1983, 1989) model, Gauvin and Hartsuiker (2020) proposed a new model of verbal monitoring for speech production. Almost simultaneously, other scholars (e.g., Broos et al., 2016, 2019) began to contextualize the model in second language acquisition for understanding the mechanism of verb self-monitoring in L2 on the grounds that Levelt’s model is originated from and underpinned by L1 speech production. Indeed, despite these progressing research efforts and the assumed differences between L1 speech production and L2 speech production (see Broos et al., 2016, for a review), major L2 speech production models (e.g., De Bot, 1992; Poulisse and Bongaerts, 1994; Kormos, 2006, 2011) are proposed mainly in light of Levelt’s (1989) L1 speech production model (Bygate, 2011; Lambert et al., 2021), or the extended version of the perceptual loop model of self-monitoring, as Levelt (1989) himself termed.

According to Bygate (2011), major L2 speech production models agree on the four main stages of the speaking process: conceptualization, formulation, articulation and monitoring throughout the stages. Conceptualization includes an access to long-term memory, tracking discourse, tracking the interlocutor’s knowledge and expectations, the overall pragmatic purpose, and the specific pragmatic-conceptual contents of a speaker’s utterances. In the stage of formulation, a speaker will encode the message from conceptualization linguistically involving lexico-grammatical selection, sequencing and phonological priming. Articulation indicates a speaker’s physical processing of the segmental and super-segmental message generated through formulation. Monitoring engages in all of the stages of a speaking process in both the covert and overt forms. Clearly, the mechanism of monitoring involved in L2 speech production has many similarities with Levelt’s (1983, 1989) perceptual loop model of self-monitoring but with more emphasis on the indispensable part of self-monitoring in speech production. The similarities are well-illustrated by Kormos’ (2011) L2 speech production model which “has been widely applied in empirical studies on L2 speaking as the major bilingual speech production model” (Zhang et al., 2022a, p. 1). In the model, Kormos strictly followed the foregoing three-loop version of Levelt’s (1983, 1989) self-monitoring model accommodating the conceptual loop, the internal loop and the external loop, which operates in response to attentional control subject to task demand variability (see Kormos, 2011; Zhang et al., 2022a, for a review of the model). Briefly, Kormos’ model vividly exemplifies the equally critical role that self-monitoring plays in L2 speech production as the construct does in L1 speech production displayed in Figure 2.

## Monitoring in computer-delivered l2 integrated speaking testing

In spite of the widely-agreed importance of monitoring in SRL and L2 speech production, if and how the construct contributes to L2 speaking testing is still opaque. In addition to the complexity of speaking, the intricacy of language testing may be another cause of the opaqueness. As Hughes and Reed (2017) have commented, language testing is “a complex field and one that the most experienced and highly regarded experts remain challenged by” (p. 87).

In language testing research, monitoring is usually treated as one of the operational forms of strategic competence or metacognitive strategy use proposed to represent the essence of L2 testing (e.g., Bachman and Palmer, 2010; Phakiti, 2016; Purpura, 2016; Zhang et al., 2021a, 2022a,b). However, since there is no conclusive definition of strategic competence, researchers tended to take an exploratory approach in investigating the working mode of the construct in L2 testing and its effect on test performance. As a result, under the macro category of strategic competence research, monitoring was identified as working effectively in some empirical studies but was absent in others. For instance, in the very limited number of studies on monitoring in the context of L2 testing, Pan and In’nami (2015) invited 170 Taiwanese university students to perform the Test of English for International Communication (TOEIC®) practice listening test, the sister products of TOEFL iBT (the Test of English as a Foreign Language Internet based), a computer-delivered L2 testing format, and to respond the questionnaires to measure the participants’ cognitive and metacognitive strategies. Through exploratory factor analysis (EFA), repeated-measures multivariate analysis of variance (MANOVA), and analysis of variance (ANOVA), the researchers concluded that the participants’ reported strategy use had a weak effect on their test scores, accounting for only 7% of the total score variance, and in the three metacognitive strategies, monitoring and evaluation, in comparison with planning, had more influence on the participants’ listening test scores. By contrast, with analysis of the data collected *via* questionnaires and interviews by the statistical means of MANOVA, Phakiti (2016) examined the influence of 384 Thai L2 test-takers’ strategic competence on their reading test performance. He found no significant correlations between test-takers’ use of monitoring and assessing and their reading performance.

With regard to the investigations into monitoring in L2 speaking testing, as noted above, the complexity of speaking and the challenges that L2 testing may impose on such investigations have made the available literature on this specific research topic scarce. Among the scarce literature, Fernandez (2018) examined the relationship between test-takers’ strategic competence, including their use of monitoring, and test performance in the third part of the IELTS (International English Language Testing System) speaking tests by means of stimulus recall. The participants were L2 learners (*n* = 12) with diverse backgrounds. The results suggested that there were no positive correlations between the participants’ monitoring and their test performance. On the contrary, Huang (2016) investigated 244 L2 learners’ strategic competence and its correlations with their performance in a large-scale standardized English proficiency test in Taiwan. After performing two sets of the test, the participants completed a survey inventory for data collection. Statistics testing methods of EFA and structural equation modelling (SEM) were performed, and the findings showed that the participants’ use of monitoring directly influenced their test performance.

Overall, the research results on the role of monitoring in L2 testing, in particular in L2 speaking testing are mixed, which suggest that further explorations of monitoring in L2 speaking testing are merited (Seong, 2014; Zhang et al., 2021a, 2022a,b). In fact, with the increasing dominance of modern technology characterized by computer-delivered or computer-assisted L2 learning, and of integrating multiple language skills in classroom instruction, some L2 testers (e.g., Swain et al., 2009; Barkaoui et al., 2013; Zhang et al., 2021a) have begun to set their focus on strategic competence in computer-delivered L2 integrated speaking tests (see Zhang et al., 2021a, for a review of the test format). Furthermore, the on-going COVID-19 pandemic that has normalized online learning and computer-delivered L2 testing has prompted the need for more research efforts in the test format. However, to our knowledge, in total, there are only seven (Swain et al., 2009; Yi, 2012; Barkaoui et al., 2013; Zhang et al., 2021a,b, 2022a,b) empirical studies related to examining monitoring in the test format, but these studied did not investigate monitoring purposely and specifically, rather they treated the construct as one form of strategic competence. Despite this, the limited number of studies have provided some inspiring insights into monitoring and helped us develop a more comprehensive view of the construct.

An example is from Barkaoui et al. (2013) who studied the relationships between test-takers’ strategic behaviors and their test performance in TOEFL iBT integrated speaking tasks (Zhang et al., 2021a). The study was conducted with L2 (*n* = 30) learners *via* the method of think-aloud. It was found that these L2 learners did not used monitoring frequently, and monitoring had no substantial effect on the participant’s speaking performance. Another example is Zhang et al. (2021a) who embedded the investigation of L2 speakers’ strategic competence in the development of Strategic Competence Inventory for Computer-assisted Speaking Assessment through two factorial analyses (the exploratory factor analysis and the confirmatory factor analysis). Zhang and colleagues found that speakers used four metacognitive strategies comprising planning, problem-solving, monitoring and evaluating in performing TOEFL iBT integrated speaking tasks. By the same token, in studying the effects of L2 learners’ perceptions of task difficulty involved in the TOEFL iBT integrated speaking tasks on their use of planning, problem-solving, monitoring and evaluating, Zhang et al. (2021b) discovered substantial negative correlations between the learners’ perceptions of task difficulty and their use of monitoring. Recently, Zhang et al. (2022a,b) examined the effects of L2 speakers’ use of the four above metacognitive strategies on their performance in the context of TOEFL iBT integrated speaking tasks, and their examination showed that monitoring exerted no significant effect on L2 learners’ speaking testing performance.

However, the seven studies each have limitations. In the studies conducted by Swain et al. (2009), Yi (2012), and Barkaoui et al. (2013) researchers administered only one subjective instrument (think-aloud or stimulus recall), without triangulation, on a small sample size for data collection, and hence the validity and generalizability of the results may be a problem (e.g., Creswell and Creswell, 2018; Creswell and Guetterman, 2019). On the other hand, although Zhang et al. (2021a,b) avoided the problem by employing multiple instruments (inventories, self-rating scales, and semi-structured interviews) among a large sample of L2 learners, these researchers regarded monitoring as an independent variable in tackling TOEFL iBT integrated speaking tasks without taking into account the effect of the interaction between monitoring and test tasks on test performance. In L2 testing, the interaction between test-takers and test tasks is a topic of frequent emphasis, as understanding the relationship in test-takers, test tasks and test performance is a fundamental problem (Bachman, 2007), and a big challenge (Hughes and Reed, 2017) that the research field has been consistently dealt with. It is also acknowledged that without considering the interactions within the three key variables involved in L2 testing, researchers are unlikely to help reveal the essence of L2 testing (Bachman and Palmer, 2010; Ellis et al., 2019). In the recent studies, though Zhang et al. (2022a,b) examined the interactions, they did no set their focus specifically on monitoring. The lack of specific focus in previous studies on either monitoring or the interactions therefore indicated a research niche that needs filling.

## Focus of this study

The integration of the above review of the indispensable and critical roles of monitoring in SRL and L2 speech production, the mixed results concerning if and how the construct work in the context of L2 speaking testing, in particular the computer-delivered integrated L2 speaking testing, the gate-keeper role of L2 testing on L2 teaching and the interactive feature of L2 testing involving the interaction between self-monitoring and tasks not only comprehensively rationalized our study but also helped to formulate the following research questions:

**Research Question (RQ) 1**: Do L2 learners use self-monitoring in performing the computer-delivered integrated L2 speaking test tasks?

If the answer to **RQ1** is yes, then **RQ2** and **RQ3** would be formed:

**RQ2**: what is the effect of self-monitoring on L2 learners’ performance in tacking the computer-delivered integrated L2 speaking test tasks?

**RQ3**: what are the effects of the interactions between self-monitoring and tasks on L2 learners’ speaking performance in the computer-delivered integrated L2 speaking test tasks?

## Method

In general, we deployed a one-way repeated measures design through which data regarding Chinese EFL learners’ performance on TOEFL iBT integrated speaking tasks and their use of monitoring elicited by questionnaires were collected and analyzed. To create the research context of computer-delivered integrated L2 speaking tests. We employed the TOEFL iBT integrated speaking tasks because compared with other internationally recognized L2 tests (e.g., IELTS) TOEFL iBT serves as a pioneer in terms of being computer-delivered and integrating L2 skills (e.g., reading, listening and speaking) with established high validity and reliability (Huang and Hung, 2018; Frost et al., 2020). Another reason for our employment of TOEFL IBT is its alignment with China’s Standards of English Language Ability (CSE) (Ministry of Education, 2021, China), which allowed us to select suitable participants with regard to language proficiency, since our participants are Chinese EFL learners.

### Participants

We examined data generated from 95 Chinese EFL university students with a score between 425 points to 500 points in the College English Test—Band 4 (CET-4), an authoritative English language proficiency test administered purposefully on Chinese university students (Zhang et al., 2021b). With reference to the CSE, this criterion meant that the student participants had an intermediate level of English language proficiency, and met the language requirement of TOEFL iBT integrated speaking tasks (Zhang et al., 2021a). The student participants were aged between 18 and 21, and the percentage of male and female students was 38% and 62% respectively. The raters were two Chinese EFL teachers with experience in rating test-takers’ performance in TOEFL iBT integrated speaking tasks. The raters came from the two universities where we recruited the student participants. All the participants joined in our study on a voluntary basis through convenience sampling, and the sample size of the student participants met the requirements of the statistical testing procedure of HLM in our study (Raudenbush and Bryk, 2002; Creswell and Creswell, 2018).

### Measures

We measured the Chinese EFL learners’ use of self-monitoring in performing the TOEFL iBT integrated speaking tasks with the Chinese version of the Strategic Competence Inventory for Computer-Assisted Speaking Assessment (SCICASA) developed by Zhang et al. (2021a). Appendix A is the Chinese version of the inventory, and Appendix B is its English version for our intended international readership.

This inventory was used because the native language of these learners is Chinese, and it measures L2 learners’ strategic competence in the context of computer-delivered integrated L2 speaking tests with a high validity and reliability (α = 0.87). However, since strategic competence defined in the inventory is composed of four metacognitive strategies, including self-monitoring, in accordance with our research purpose, we only used the self-monitoring section of the SCICASA accommodating 7 items with a 6-point Likert scale: 0 (never or almost never use), 1 (rarely), 2 (sometimes), 3 (often), 4 (usually), and 5 (always or almost always). An example of an item is, “I knew what to do if my intended plan did not work efficiently during the task.” Apart from the items, the SCICASA also collects data about participants’ background information (e.g., English language proficiency, age and gender).

With respect to the TOEFL iBT integrated speaking tasks, given the English language proficiency of the Chinese EFL learners, and the authenticity of testing conditions L2 learners will deal with in real-world settings, we used a whole set of the new TOEFL iBT integrated speaking section for L2 learners with intermediate level of language proficiency from the test database, which includes three different tasks: Task 1, Task 2, Task 3 (ETS, 2022a). In order to maintain the validity and the reliability of the test, we used the original version of the tasks without doing any modifications (Creswell and Creswell, 2018). As our research questions did not focus on the test tasks, detailed features of the three test tasks are not presented here. For those interested in the TOEFL iBT integrated speaking task features, Zhang and Zhang (2022) provided a detailed descriptions of the old version (before the recent reform of the test that took place in 2018) of the test format; and the delineation of the new version of the test can be found in the official website of ETS (2022a).

The Chinese EFL performance was measured by the two raters with reference to the TOEFL iBT integrated speaking rubric (ETS, 2022b) which measures oral performance in accordance with four criteria (the score range for each criteria is 0 to 4 points): Delivery (e.g., fluency, clarity, and pronunciation); language use (e.g., grammatical accuracy and vocabulary use); topic development (e.g., cohesion and progression of ideas), and general description.

### Data collection

We collected data in multi-media lecture rooms where the Chinese EFL learners performed the three test tasks on computers installed with the TOEFL iBT integrated speaking practice online database software. Each time the learners finished a task, they were invited to answer the SCICASA by ticking a number for each item that represents the frequency of their monitoring use. For example, if they believed that they often used monitoring reflecting by the item of “I knew what to do if my intended plan did not work efficiently during the task,” they would ticked the number of 3. The questionnaire was delivered to the learners through a Chinese online survey platform Wenjuanxing (2021) so that learners used their mobile phones to answer the inventory for convenience and research efficiency (Dörnyei and Taguchi, 2009). To counterbalance the carry-over and the order effects that might be caused by the one-way repeated measures design, we sequenced the three test tasks in a Latin-square design and provided the learners with around 10 min break between tasks, as suggested by some scholars (e.g., Weir et al., 2006; Verma, 2015; Corriero, 2017). Every learner’s responses to the test tasks were recorded automatically by the TOEFL iBT integrated speaking practice online database software in a single file named in accordance with the learners’ codes given by themselves for anonymity in order to protect their privacy, and for identifying the files for the purpose of the following data analysis. These files were then dispatched randomly to the two raters for scoring (Weir et al., 2006).

To reach intra-rater and inter-rater agreements, we invited the two raters to join in our training programs in line with Huang and Hung (2013). After the training, the indexes of the intra-rater reliability and inter-rater reliability were all above the thump-up value (> 0.70; Frey, 2018; Teng, 2022). Regarding the scoring method, the two raters employed the analytic scoring before holistic scoring. In detail, they first scored independently the four segments of each participant’s oral performance by referring to the rubric with a score ranging from 0 to 4 points given to each segment. As a result, four scores for the four segments were obtained. Then, the raters aggregated the four scores for a composite score. Next, the composite scores from the two raters for the three tasks were aggregated before being divided to generate an average score, a holistic score to statistically measure the participants’ general oral performance (Huang and Hung, 2013).

During data collection, we addressed ethics issues by strictly following the guidelines set by the relevant departments responsible for human participants ethics in the universities where we recruited the Chinese EFL learners and the raters (Creswell and Creswell, 2018). For instance, before the study was conducted, the researchers contacted the universities where we recruited the participants for official permission. A consent form was provided in which the purpose of our study and the information indicating that participants were voluntary and that the study would never place them at undue risks were clearly stated. All the participants were provided with a form on participation and a consent form for their signatures. They were also entitled to ask for unconditional destroy of the data collected on them at their will. All participants were given a small gift worth around 100 CNY with a thank-you letter as a token of gratitude.

### Data analysis

Descriptive analysis was deployed for attaining the means of the Chinese EFL learners’ use of self-monitoring in tackling the three test tasks to address RQ1, and for the means of these learners’ speaking performance indicated by their test scores (Barkaoui et al., 2013; Ellis et al., 2019), which was used for the subsequent analysis for answering RQ2 and RQ3. In conducting the descriptive analysis, we did the required assumption tests, including inspecting the normality of the data by examining the values of the standard deviation, the skewness and the kurtosis (Pallant, 2016).

To address RQ2 and RQ3, we established two hierarchy linear models with each composed of two levels through the statistical testing procedure of HLM, which is advocated to investigate data on performance collected in testing conditions through a one-way repeated measures (Barkaoui, 2013, 2015; In’nami and Barkaoui, 2019), as was the case with our study. In the two models, one was a full model in which the first level (Level-1) was for the three TOEFL iBT integrated speaking tasks, and variables at this level were the Chinese EFL learners’ test scores, the outcome variables, and the three test tasks were the predictor variables. The second level (Level-2) was for the Chinese EFL learners, and variable at this level was the predictor variable of the learners’ use of self-monitoring (Barkaoui, 2013, 2015; In’nami and Barkaoui, 2019). According to Raudenbush and Bryk (2002), in order to examine the effects of the interactions between self-monitoring at Level-2 and test tasks at Level-1 on test scores, the focus of building and the following running the HLM was to check the cross-level interactions, which determined the methods in which we entered the data into the two models: data on self-monitoring were grand-mean centred before being entered the model, and data on test tasks were entered into the model as dummy variables in accordance with the *k*-1 formula commonly used for coding dummy variables. In consequence, we treated Task 1 as the baseline, and generated two dummy variables: Task 2 and Task 3. In coding the dummy variables, we used 1 to indicate the specific task when it was performed, and 0 to denote an undone task. For instance, if a Chinese EFL learner was doing Task 2, data on Task 2 were labelled as 1 and data on Task 3 were represented by the number of 0. Likewise, if a learner was doing Task 3, this task was marked as 1, and data on Task 2 were referred to as 0. Since test tasks were treated as dummy variables, we entered the task data as uncentered (Barkaoui, 2013, 2015; In’nami and Barkaoui, 2019).

The other model was the null model in which there were no predictor variables. We built the model for the Intra-class Coefficient (ICC) to evaluate if it was appropriate to administer HLM on our data. In addition, ICC can also be used for model fit examination with its value being close to 1 suggesting a good model fit. In model evaluation, we mainly referred to deviance statistics (a smaller value denotes a better model fit), and significance tests: *t*-tests for the fixed effects (*p* < 0.05) of the testing parameters, and the random effects of which were assessed *via* Chi-square tests (*p* < 0.05). Moreover, our assessment of model fit also included the examination of the reliability of Level-1 random coefficient, and our visual inspection of the normality of residuals of Level-1 and Level-2 with reference to Q-Q plots and scatter plots. In running the models, we deployed the estimation method of the Fully Maximum Likelihood (see Barkaoui, 2013, 2015; Zhang et al., 2022b, for detailed application of the HLM in L2 testing conditions).

## Results

Descriptive analysis revealed that data on both self-monitoring and speaking performance demonstrated normal distribution with values of the skewness and the kurtosis meeting the required threshold of normality (−3 ≤ skewness ≤3; −8 ≤ kurtosis ≤8; Pallant, 2016; Frey, 2018). Based on the assumption testing results, we further examined the means of the Chinese EFL learners’ use of self-monitoring and their speaking performance across the three integrated L2 speaking testing tasks as shown by Table 1. Means of self-monitoring ranged from 3 to 3.30. With reference to the SCICASA where 3 suggests “often” and 4 indicates “usually,” it is obvious that in performing the three tasks, the Chinese EFL learners used self-monitoring quite often. This result answered RQ1 on if L2 learners use self-monitoring in performing the computer-delivered integrated L2 speaking test tasks.

**Table 1:** Descriptive analysis of self-monitoring and test scores across tasks.

Tasks	Self-monitoring		Test scores	
	Means	SD	Means	SD
Task 1	3.16	0.88	5.45	2.65
Task 2	3.21	0.87	4.40	2.95
Task 3	3.30	0.89	4.40	2.95

SD = standard deviation.

The means of the test scores were used to build the two HLM models for addressing RQ2 and RQ3. Table 2 demonstrates the results of the null model and the full model based on our model fit evaluation.

**Table 2:** Results of the null model and the full model.

	Null model	Full model
Fixed effects		
Level1coefficient(r)	3.05	
$\text{Intercept}\;\left(\gamma_{{\;}_{00}}\right)\;\left(sig.\right)$	4.88(0.00)	5.20(0.00)
$\text{Task}\;2\;\left(\gamma_{{\;}_{10}}\right)\;\left(sig.\right)$		−0.96(0.00)
$\text{Task}\;3\;\left(\gamma_{{\;}_{20}}\right)\;\left(sig.\right)$		−0.50(0.00)
Level2coefficient(sig.)		
$\text{Self-monitoring}\;\left(\gamma_{{\;}_{01}}\right)$		0.10(0.51)
Cross−level interaction coefficient(sig.)		
$\text{Self-monitoring in Task}\;2\;\left(\gamma_{{\;}_{11}}\right)$		0.21(0.48)
$\text{Self-monitoring in Task}\;3\;\left(\gamma_{{\;}_{21}}\right)$		−0.17(0.57)
Random effect		
$\text{Between}-\text{students variance}\;(\mu_{{\;}_{0}})$ (sig.)	5.32(0.00)	5.34(0.00)
$X^{2}\;\left(\text{df}\right)$	592.03(94)	640.00(93)
ICC	0.63	
Reliability	0.84	0.85
Modelfit		
Deviance(parameters)	1298.60(3)	1280.94(8)

In the table, γ_01_ referred to the fixed effects of self-monitoring reported by the Chinese EFL learners on the average mean of their oral scores across the three tasks, while μ_0_ indicated the random effects of the learners’ heterogeneity, including their use of self-monitoring, on the mean of their oral scores across the tasks that could not be explained in the two models. Furthermore, γ_11_ and γ_21_ denoted the cross-level effect or the effect of the respective interactions between self-monitoring and Task 2 and between self-monitoring and Task 3 on the Chinese EFL learner’s oral scores. These indices were the research foci of this study as demonstrated by the research questions.

From Table 2, it can be seen that in the null model, the value of ICC is 0.63, meaning that 63% of the total variance in the Chinese EFL learners’ oral scores was accounted for by their individual differences, including their use of self-monitoring, at Level-2, whereas tasks at Level-1 explained about 37% of the total variance in the scores. The result indicated the necessity and appropriateness of running HLM on the current data set for addressing RQ2 and RQ3 (Raudenbush and Bryk, 2002; Weng, 2009). Moreover, the reliability estimate in the null model for the learners’ mean oral scores across the three computer-delivered integrated speaking tasks was 0.84, suggesting that almost 85% of the variation in each learner’s oral scores across the speaking tasks was potentially explicable by individual level or Level 2 predictors. The deviance value of the null model was 1298.60 which was used in the subsequent model comparisons for model fit evaluation (Raudenbush and Bryk, 2002; Weng, 2009).

As for the full model, Table 2 shows that the coefficient for self-monitoring (γ_01_) was 0.10, with its *p* value being 0.48, much larger than 0.05, the cut-off rule value. This result suggested that variances at Level-2 in the Chinese EFL learners’ use of self-monitoring had no direct and substantial effects on their oral scores across the three tasks. Likewise, the *p* values of γ_11_ (0.48), γ_21_(0.57), the coefficients denoting the respective effects of cross-level interactions between self-monitoring and Task 2 and between self-monitoring and Task 3 on the learners’ oral scores were both greater than 0.05, indicating that the interactions between Chinese EFL learners’ reported use of self-monitoring and test tasks did not have statistically significant effects on their oral scores. The result revealed that self-monitoring did not affect oral scores on Task 1, either, given the fact that Task 1 was regarded as the baseline task as a way of coding dummy variables. In addition, the value of reliability in the full model was 0.85, suggesting that Level-2 individual differences accounted for 85% of the variance in the Chinese EFL learners’ oral scores at Level-1.

Of note, although the effects of tasks on oral scores were not the focus of this study, we reported them in Table 2 for a comprehensive interpretation of our research results. In Table 2, values in the two model revealed that the *p* values of γ_00_ (0.00) which referred to the average means of the Chinese EFL learners’ oral scores across the three integrated speaking tasks in the null model and the full model were below value 0.05, indicating that significant variance in the mean scores across tasks and individuals existed. Similarly, the *p* values of γ_10_ (0.00) and γ_20_ (0.00) which represent the respective effects of Task 2 and Task 3 on oral scores were both smaller than the threshold value of 0.05, meaning that the two tasks had substantial effects on the learners’ oral scores. Accordingly, the baseline task, Task 1 also had considerable impact on oral scores (Raudenbush and Bryk, 2002; Weng, 2009).

In model fit evaluation, we compared the ordinary standard errors and the robust standard errors. The comparison showed that there was no significant variance, and hence, model specification was acceptable. Additionally, the decrease in the values of deviance from 1298.60 in the null model to 1280.94 in the full model demonstrated an improvement of model fit. Finally, the investigation into Level-1 random coefficient reliability (γ _00_ = 0.85, large than.05, the thumb-up rule value) and the visual inspection of the Q-Q plots and scatter plots of the residuals for both Level-1 and Level-2 showed that the full model fitted well the current data set (Raudenbush and Bryk, 2002; Weng, 2009; Barkaoui, 2013, 2015).

## Discussion

The current study investigated if and how self-monitoring, the core SRL skill (Winne and Hadwin, 1998; Winne, 2011, 2018), operates in the context of computer-delivered integrated L2 speaking testing in a multi-disciplinary approach. Our data analysis revealed two major results: first, Chinese EFL learners used self-monitoring quite often in performing the three tasks. Second, self-monitoring, either working in an independent manner or working through its interactions with tasks, had no substantial effects on the Chinese EFL learner’s oral performance. The two results are seemingly contradictory to each other but understandable.

During performing the speaking tasks, the Chinese EFL learners’ active use of self-monitoring might have to do with their preparations for the tasks. According to Bygate (2018), Lambert et al. (2021), and Robinson (2010), effective preparations before tasks may provide L2 speakers with support in their pre-conceptualizing and pre-formulating messages, allowing them to depend on the conceptualized content which they rehearsed previously and the linguistic resources that they have activated recently during speech production. Therefore, the more support L2 speakers attain from task preparations, the less self-repair they will tackle in their initial encoding of the utterance. The correlation in a negative direction between task preparations and self-monitoring in terms of content, language and chances of practice reflects the accounts of attention to self-monitoring proposed by Kormos (2011) and Lambert et al. (2017), which indicate that when a L2 speaker has limited access to support through the means of task preparations, her need for self-monitoring would be expected to increase. This was very likely true of this current study where 98% of the Chinese EFL learner participants reported in the background information of the SCICASA that it was the first time for them to attend the TOEFL iBT integrated speaking tasks. This showed that the student participants had no experience in task preparations for the test. With few task preparations, it is accountable that their need for self-monitoring during L2 speech production increased, and accordingly their use of this strategy in performing the three TOEFL iBT integrated speaking tasks demonstrated a high frequency. The frequently reported use of self-monitoring by the Chinese EFL learners also supports prior studies reviewed earlier such as Pan and In’nami (2015), Zhang et al. (2021a, 2022a,b) in which self-monitoring displayed a high frequency in L2 learners.

In essence, content, language and practice, the three fundamental factors involved in task preparations denote L2 learners’ knowledge of tasks in SRL and the task demands imposed on L2 speakers during L2 speech production. This task-dependent feature of self-monitoring has been evidenced by a large volume of literature, especially in the research field of language testing (e.g., Barkaoui, 2015; Youn and Bi, 2019).

Contrary to our initial expectations, our study identified that the interactions between self-monitoring and test tasks had no significant effects on the Chinese EFL learners’ speaking performance. Taking into account the fact that the Chinese EFL learners’ oral scores were considerably affected by tasks, as shown by the results of the HLM in Table 2, the conflict between the actual effects of the interactions and the assumed effects was possibly caused by the learners’ use of self-monitoring which had no significant effects on L2 speakers’ performance when the construct was working independently. The lack of functioning displayed by self-monitoring in affecting Chinese EFL learners’ performance in the computer-delivered testing context is also likely due to the severe time pressure on the speakers imposed by the testing context (Levelt et al., 1999; Oomen and Postma, 2001; Ganushchak and Schiller, 2006). It is known that L2 tests are usually given under limited time conditions, as is the TOEFL iBT integrated speaking tasks which required the Chinese EFL learners to finish the speaking tasks in 1 min based on their understanding of a short reading passage and/or a short listening material with the reading and listening also being done in a limited time (Bachman and Palmer, 2010; Zhang and Zhang, 2022; Zhang et al., 2022a,b). Under the severe time pressure, the Chinese EFL speakers were assumed to speak fast in order to finish the test tasks within the time limit (Ganushchak and Schiller, 2006). In such situations, a speed-accuracy trade-off effect might occur on them, and consequently, they were very likely to shift between speed and accuracy, a process that is proposed to be controlled through self-monitoring (Levelt et al., 1999; Oomen and Postma, 2001; Ganushchak and Schiller, 2006). As verbal self-monitoring is a controlled resource-limited process, when time pressure was present, it is natural that time, as the key resources in performing tasks in any forms (Robinson, 2010; Ellis et al., 2019), available to the Chinese EFL speakers were not sufficient for them to shift between speed and accuracy in an optimal manner *via* self-monitoring, and hence it was possible that the speakers had to allocate their limited time for the monitor based in the conceptualizer (Levelt et al., 1999; Oomen and Postma, 2001; Ganushchak and Schiller, 2006). In coping with the time pressure, the monitor might not have enough time to detect the possible errors in the encoded phonetic plan generated in the formulator in the internal loop, and to inspect the correctness and appropriateness of the Chinese EFL speakers’ utterance in the external or the auditory loop. By the same token, time allocated for self-monitoring the appropriateness taking place in the conceptualizer in the conceptual loop might also be negatively influenced. However, as the monitor was located in the conceptualizer, closer to the conceptual loop in comparison with the other two loops, it was predicted that the negative influence of time pressure on the conceptual loop in the Chinese EFL learners’ speech production was the weakest in the three loops (Oomen and Postma, 2001). Yet, in general, from this perspective of resource allocation (Levelt, 1989; Levelt et al., 1999; Oomen and Postma, 2001; Ganushchak and Schiller, 2006), it is explainable that under the time pressure caused by the TOEFL iBT integrated speaking tasks, the Chinese EFL learners’ verbal self-monitoring did not function positively. Therefore, it might not exert significant effect on the learners’ speaking performance despite the fact that these learners actively employed self-monitoring in order to finish the given testing tasks with the required time range. Among the extant empirical studies of relevance that we reviewed above, Swain et al. (2009), Barkaoui et al. (2013), and Zhang et al. (2022a,b) all found that self-monitoring had no considerable influence on L2 speakers’ performance, and they agreed that time pressure might account for such a result.

Another possible cause of the lack of functioning associated with verbal self-monitoring has to do with the Chinese EFL learners’ motivation. Motivation is a complex and multidimensional goal-driven activity. It is one of the most important individual factors that impacts L2 learners’ strategy use, including self-monitoring, which has been empirically supported by researchers (e.g., Oxford, 2017; Cohen, 2018; Zhang et al., 2021b). In the well-accepted dichotomy of motivation in L2 learning (Deci and Ryan, 2000; Dörnyei, 2019), intrinsic motivation refers to learners’ behaviors that can bring them gratification without thinking about the consequence of their behaviors such as learning a language for the joy of learning *per se*. In contrast, extrinsic motivation, another constituent of the dichotomy, functions like a stimulus in learners’ learning process through which learners can receive external rewards such as getting an ideal job or being admitted to a university. Existing literature (e.g., Ganushchak and Schiller, 2008; Maruo et al., 2016) on speech production has documented the relationship between an individual’s extrinsic motivation and verbal self-monitoring: in high extrinsic motivation conditions where the detection and repair of errors by L2 learners’ self-monitoring are closely linked to their monetary loss or penalty, L2 learners tend to use their self-monitoring in an effective and efficient way for fewer errors (Boksem et al., 2006). On the other hand, if L2 speakers’ performance has nothing to do with reward or punishment, they typically pay little attention to using self-monitoring, and so their verbal self-monitoring usually functions passively. In this study, since the Chinese EFL learners were volunteers, it is obvious that they did not participate in the integrated speaking tests for monetary gain. As a result, if they did not perform well, they did not need to face penalty or punishment. In such circumstances, it is justifiable to think that these learners were generally unmotivated extrinsically or alternatively put, they were attending the test in low extrinsic motivation conditions which commonly bring about a lack of functioning of self-monitoring (Zhang et al., 2021b). This may explain why the Chinese EFL learners’ speaking performance was not substantially affected by their verbal self-monitoring.

In fact, as illustrated in Figure 1, in SRL, motivation also influences the functioning of self-monitoring, and the consistency of the role of motivation in both SRL and the TOEFL iBT integrated speaking tasks suggests the possibility of providing the wash-back effect of understanding monitoring in L2 testing on teaching and learning effective use of self-monitoring in L2 classroom instructions through this study, which evidenced the pedagogical contributions we aimed to make, as presented in the following section.

## Contributions and limitations

This study shows that despite the active use of self-monitoring reported by the Chinese EFL learners, the construct had no significant influence on their performance in computer-delivered testing. The study, therefore, does not corroborate the facilitating role of self-monitoring in determining performance in SRL and L2 speech production in non-testing conditions. This inconsistency indicates that self-monitoring did not work in L2 speaking testing contexts. Hence, it is proposed that in addition to teaching self-monitoring in normal learning settings, L2 teachers should purposefully create specific learning settings in their classroom instructions, which permit their students, the L2 learners, to rehearse the SRL skill of self-monitoring in more authentic testing conditions. Further, L2 teachers integrate their teaching of self-monitoring in such testing contexts into their syllabus in an appropriate percentage for their instructions, in particular, L2 speaking instructions, so that L2 learners can have frequent opportunities to polish their self-monitoring skill in tackling authentic tests. In this way, L2 learners can not only practice how to use the SRL skill effectively in normal learning situations but also in high-stakes testing situations. Hopefully, this would empower the L2 learners to switch smoothly and competently across contexts in using self-monitoring for achieving real learner autonomy, one of the fundamental goals of SRL. Otherwise, it is very possible that self-regulating learners, though being taught sufficiently to equip themselves with the self-monitoring skill, are only capable of using the skill in their routine learning conditions with which they are familiar without being able to perform as well as we expect of a self-regulating learner, a term usually associated with a good performer in various contexts, including L2 tests. This possibility echoes the famous quote from Wattles (1910): “It is essential to have good tools, but it is also essential that the tools should be used in the right way” (p. 119).

Additionally, the active use of self-monitoring elicited by the TOEFL iBT integrated speaking tasks suggests that L2 teachers can borrow directly from the test format or tests of the same sort in preparing tasks for their pedagogical purpose of teaching their students how to use the SRL skill for L2 speaking, particularly computer-delivered L2 speaking and or testing in their daily classroom instructions. Such task preparation practice is assumed to enable L2 teachers to competently cope with the COVID-19 times, one of the biggest challenges in education at the moment, which has imposed increasing demand on online learning and testing (Zhang, 2021). On L2 learners’ side, the authenticity of integrated speaking tasks in duplicating the real-world settings (Thomas, 2019) will help to familiarize them with the authentic language use tasks they may deal with for SRL learning beyond classroom settings. As L2 learners’ familiarity with tasks or their prior knowledge of tasks is one of the components of cognitive conditions in the SRL model shown by Figure 1, the use of the integrated speaking tasks in classroom instruction will benefit L2 learners regarding how to be self-regulating learners.

Apart from the pedagogical implications, the working mode of self-monitoring discovered in our study is also expected to provide additional empirical evidence for the operational mechanism of the construct underpinned by Levelt’s (1983, 1989) perceptual loop model of self-monitoring in L1 speech production and Kormos’ (2011) bilingual speech production model, as stated earlier. In the meanwhile, the discovery will offer some insights into the definitions and taxonomies of strategic competence, a research topic under debate that needs further exploration in the field of L2 testing where self-monitoring has been proposed as one of the constituents of strategic competence with mixed empirical evidence (Zhang et al., 2022a,b), as reviewed previously.

Despite the pedagogical and theoretical contributions of this study, it is necessary to point out its limitations. First, because of the convenience sampling that we employed to recruit student participants, the L2 learners in this study were all Chinese EFL university students with similar language proficiency, age range and EFL learning experiences. The sampling homogeneity may reduce the generalizability of our research results in other contexts where L2 learners may not be Chinese EFL learners or Chinese EFL learners with similar characteristics to those in this study (Creswell and Creswell, 2018). Second, we investigated if and how self-monitoring worked in computer-delivered integrated L2 speaking testing, but we did not explore why the construct worked in the way as revealed by our investigation *via* the employment of interviews, think-aloud protocols and self-reflections as some researchers (e.g., Creswell and Creswell, 2018) proposed. For a comprehensive understanding of self-monitoring, it is suggested that in future research of similarity, it is merited to study not only “if” and “how” but also “why” through adoption of the aforementioned research methods.

## Data availability statement

The raw data supporting the conclusions of this article will be made available by the authors, without undue reservation.

## Ethics statement

The studies involving human participants were reviewed and approved by relevant departments responsible for human participants ethics in Anhui University of Finance and Economics, Bengbu, Anhui Province, China, and Bengbu University, Bengbu, Anhui Province, China. Participants provided their written consent forms to participate in this study.

## Author contributions

WZ conceived of the initial idea, fine-tuned by AW. WZ designed the study, collected, analyzed the data, and drafted the manuscript. AW revised and proofread the manuscript. WZ finalized and submitted the manuscript. All authors contributed to the article and approved the submitted version.

## Funding

This study was funded by the Research Grant for New Researchers by Quzhou University (Project Number: BSYJ202205), and the Project co-worked by SISU Education Group and Quzhou University (Project number: H2023006).

## Conflict of interest

The authors declare that the research was conducted in the absence of any commercial or financial relationships that could be construed as a potential conflict of interest.

## Publisher’s note

All claims expressed in this article are solely those of the authors and do not necessarily represent those of their affiliated organizations, or those of the publisher, the editors and the reviewers. Any product that may be evaluated in this article, or claim that may be made by its manufacturer, is not guaranteed or endorsed by the publisher.
