# Supplementary material for: From self-regulated learning to computer-delivered integrated speaking testing: Does monitoring always monitor?
Source: Front Psychol. 2023 Feb 1;14:1028754. doi: 10.3389/fpsyg.2023.1028754 (PMC9928721; doi:10.3389/fpsyg.2023.1028754)
Supplement: Supplementary file 1 [file Data_Sheet_1.docx]

**Appendix A**

**计算机辅助综合口语测试任务中中国英语学习者策略能力量表**

**Chinese Version**

**第一部分**

请在下面的方框中打钩(√)或在空白处填写您的回答以方便我们更好地了解您的问卷回答

1． 个人代码

2． 年龄

3． 性别 ： 男_ □ 女_ □

4． 到目前为止您已经学习英语学习多长时间：

7~9 年_ □ 10~12_□ 年 13~15 年_□ 其他---------

5.参加过何种英语考试

CET4_□ CET6_□ BEC_□ IELTS_□ TOEFL_□

**第二部分**

请认真阅读表格中的内容并打钩(√)选出你在综合口语考试中有哪些想法： 0 (从不或几乎不), 1 (很少), 2 (偶尔), 3 (常常), 4 (大多情况下), 5 (总是或几乎总是这样)

| **您的想法** | **0** | **1** | **2** | **3** | **4** | **5** |
| --- | --- | --- | --- | --- | --- | --- |
| 1.我清楚题目要求我做什么。 |  |  |  |  |  |  |
| 2.我明白需要规划答题过程。 |  |  |  |  |  |  |
| 3.我想过需要做什么才能完成任务。 |  |  |  |  |  |  |
| 4.我确信已清楚任务目标 |  |  |  |  |  |  |
| 5.我明白完成任务所需要的主要步骤。 |  |  |  |  |  |  |
| 6.我事先组织好了想说的内容的结构。 |  |  |  |  |  |  |
| 7.我会借助已有的知识（如单词的上下文，构词及话题） 来猜测陌生单词或词组的意思。 |  |  |  |  |  |  |
| 8.我根据上下文猜测话题。 |  |  |  |  |  |  |
| 9.我借助已有知识来完成话题任务。 |  |  |  |  |  |  |
| 10.(口语表达)想不起来某个英语单词时，我用相通常思的其他词或词组。 |  |  |  |  |  |  |
| 11.我使用近义词之类其他方法来表达意思。 |  |  |  |  |  |  |
| **您的想法** | **0** | **1** | **2** | **3** | **4** | **5** |
| 12.我知道答题时何时该加快速度。 |  |  |  |  |  |  |
| 13.我知道答题中何时要更加仔细。 |  |  |  |  |  |  |
| 14.答题过程中我知道自己用掉多少时间。 |  |  |  |  |  |  |
| 15.口试过程中，我知道自己哪些地方说得比较地道 |  |  |  |  |  |  |
| 16.我能把题中的信息与己有知识联系起来。 |  |  |  |  |  |  |
| 17.答题过程中我会记录重要词语与概念。 |  |  |  |  |  |  |
| 18.如果进展不顺利，我知道该怎么对付。 |  |  |  |  |  |  |
| 19.完成口语任务后我在脑子里给自己的表现打了个分数。 |  |  |  |  |  |  |
| 20.任务结束后我检查自己是否达到目标。 |  |  |  |  |  |  |
| 21．考试后我会检查自己的错误。 |  |  |  |  |  |  |
| 22.我会评价自己在答题表现方面的满意度。 |  |  |  |  |  |  |
| 23.我会评价原定计划实施的有效性。 |  |  |  |  |  |  |

**Appendix B**

**The Strategic Competence Inventory for EFL Learners in Computer-assisted Integrated Speaking Tests**

**English Version**

**Part One**

Please provide your information by ticking (√) in the box or write your responses in the space so we can better understand your answers.

1. Code:
2. Age:
3. Gender: Male_□ Female_□ Gender diverse_□
4. The years you have been learning English to present:

7~9 years_ □ 10~12 years_□ 13~15 years_□ Others_______.

1. English proficiency reflected by test

CET4_□ CET6_□_ BEC_□ IELTS_□ TOEFL_□

**Part Two**

Please read each of the following statement and indicate how you thought during the integrated speaking test by ticking (√) 0 (never), 1 (rarely), 2 (sometimes), 3 (often), 4 (usually), and 5 (always)

| **Your thinking** | **0** | **1** | **2** | **3** | **4** | **5** |
| --- | --- | --- | --- | --- | --- | --- |
| 1. I knew what the task questions required me to do. |  |  |  |  |  |  |
| 2. I was aware of the need to plan a course of action. |  |  |  |  |  |  |
| 3. I thought about what to do to complete the task well. |  |  |  |  |  |  |
| 4.I made sure I clarified the goals of the task |  |  |  |  |  |  |
| 5. I understood the essential steps needed to complete the task. |  |  |  |  |  |  |
| 6. I organized the structure of what I was going to say before speaking. |  |  |  |  |  |  |
| 7. I guessed the meaning of the unknown words or expressions by using my knowledge (e.g. words in the context, knowledge of word information, knowledge of the topic. |  |  |  |  |  |  |
| 8. I used the context to guess the topic. |  |  |  |  |  |  |
| 9. I drew on my background knowledge to complete the task. |  |  |  |  |  |  |
| 10. I made up new words or guess if I didn’t know the right ones to use. |  |  |  |  |  |  |
| 11. I used a word or phrase that means the same thing when I could not think of a word in English. |  |  |  |  |  |  |
| 12.I knew when I should complete a task more quickly. |  |  |  |  |  |  |
| 13. I knew when I should complete a task more carefully |  |  |  |  |  |  |
| 14. I knew how much time had gone by. |  |  |  |  |  |  |
| 15. When I was speaking, I knew when I had spoken in a way that sounded like a native speaker. |  |  |  |  |  |  |
| 16. I related the incoming information to what I had known. |  |  |  |  |  |  |
| 17.When I was performing my task, I took notes on the important words and concepts. |  |  |  |  |  |  |
| 18. I knew what to do if my intended plan did not work efficiently during the task. |  |  |  |  |  |  |
| 19. I mentally give myself a grade after I finished my task. |  |  |  |  |  |  |
| 20. I checked whether I had accomplished my goal after completing my task. |  |  |  |  |  |  |
| 21. I checked the mistakes I had made in the task. |  |  |  |  |  |  |
| 22. I evaluated my performance satisfaction as I moved along the task. |  |  |  |  |  |  |
| 23. I evaluated whether my intended plans worked effectively. |  |  |  |  |  |  |
